# Is the Meckel diverticulum still a bad boy in general surgery? Case report of an intestinal obstruction managed through a single-port access and review of the literature

**DOI:** 10.1093/jscr/rjaa584

**Published:** 2021-01-25

**Authors:** Gabriele Bauci, Walter Kolb, Joanna Janczak

**Affiliations:** Department of General, Visceral, Endocrine, and Transplant Surgery, Kantonsspital St. Gallen, St. Gallen, Switzerland; Department of General, Visceral, Endocrine, and Transplant Surgery, Kantonsspital St. Gallen, St. Gallen, Switzerland; Department of General, Visceral, Endocrine, and Transplant Surgery, Kantonsspital St. Gallen, St. Gallen, Switzerland

**Keywords:** Meckel Diverticulum, NET, endocrine surgery

## Abstract

In our institution single-port diagnostic laparoscopy is the routine procedure for patients with acute abdominal emergencies. Here, we present a case of intestinal obstruction due to a torqued Meckel diverticulum successfully managed through a single-port incision.

## INTRODUCTION

Meckel diverticulum (MD) is a congenital anomaly with a prevalence of around 2% in the general population. The incidence, a cancer in the MD, is reported to be about 1.44 per 10 million people [1]. German anatomist Johann Meckel first described the embryologic origin of this anomaly. Up to 60% of patient with an MD harbours a malignancy but only 20 to 30% of these lesions will be symptomatic [1]. The majority of neoplasms are neuroendocrine tumours (NETs), around 77%. In daily clinical practice the surgeon must approach the case basing the decisions on many factors such as age, co-morbidities, localization and personal skills. Until now there is no strong consensus on the optimal surgical technique. Herein we report an unusual case of MD leading to a mechanical ileus. This report describes the easy and feasible management of intestinal obstruction due to an MD using a single-port incision. In our opinion, this minimally invasive approach can be very useful to perform a variety of procedures.

## CASE REPORT

**Figure 1:**
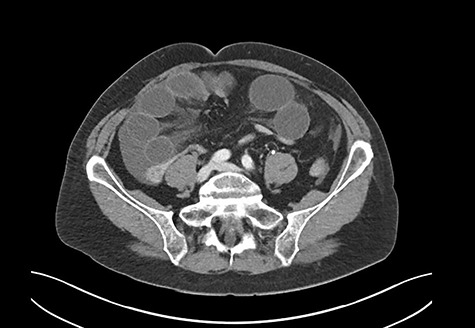
CT scan: severe mechanical obstruction of the small intestine caused probably by adhesions.

A 69-year-old woman was referred to our institution due to clinical signs of acute abdomen. She reported diffuse abdominal pain without an identifiable trigger, associated with recurrent vomiting. The patient had no history of abdominal operations, took no medications, and never experienced a similar episode before. A computed tomography (CT) scan revealed small bowel ileus (Fig. 1). The radiologist suspected a mechanical obstruction.

**Figure 2:**
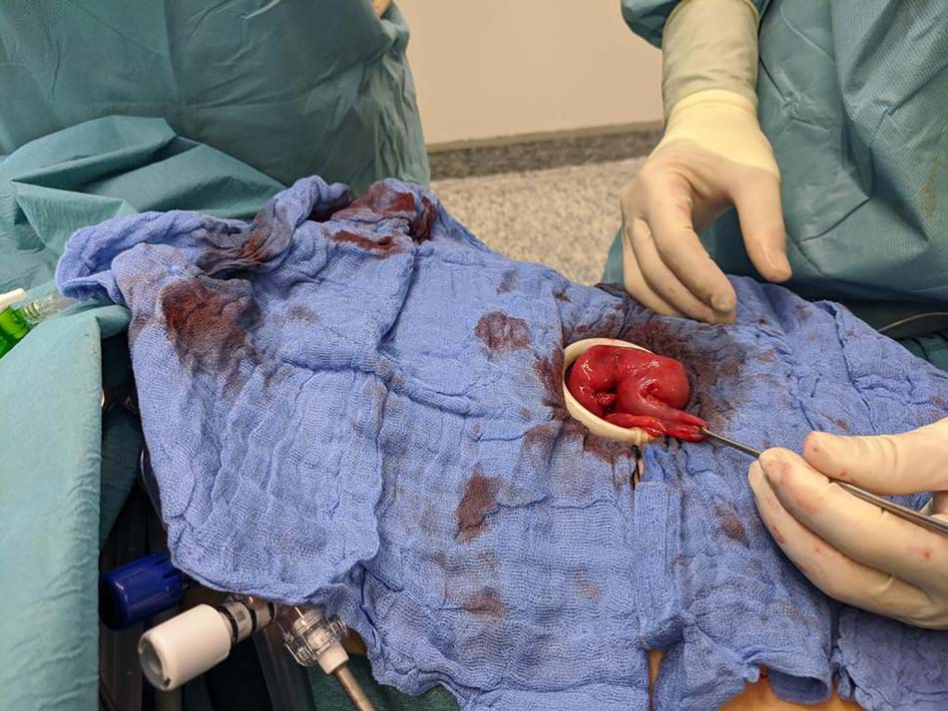
Multipurpose using of the single port.

**Figure 3:**
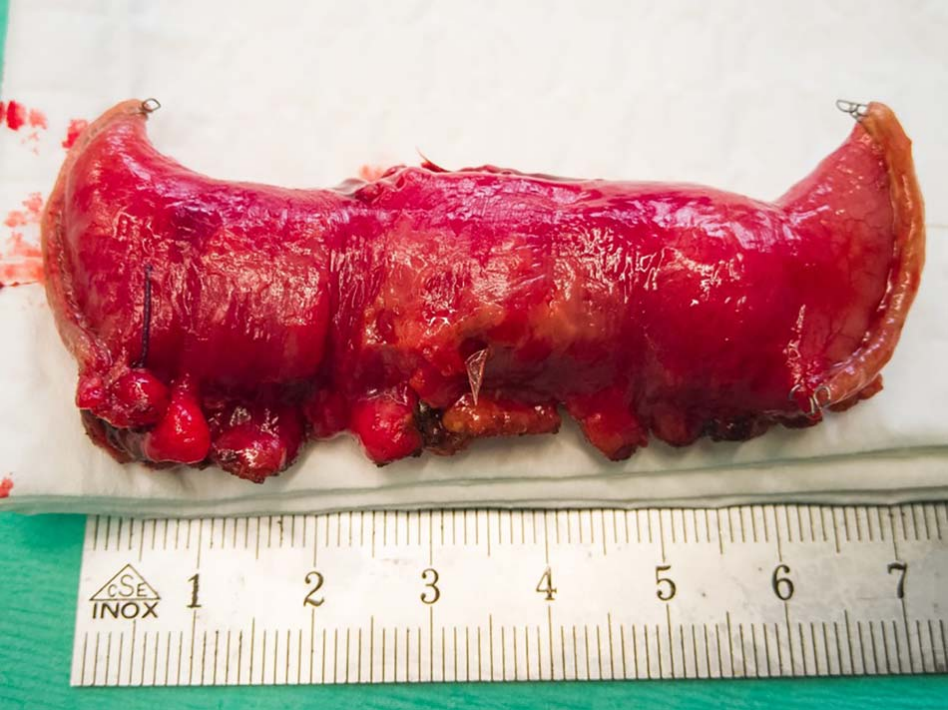
Small bowel resection.

This gave the indication for the operative exploration. As usual, we performed a single-port laparoscopy to assess the problem and decided the further procedures. A not-inflamed, 3 cm big MD with strong perifocal fibrotic adhesions with the small bowel was discovered 1 m proximal to the ileocecal valve. We continued the operation by using the single incision as a mini-laparotomy (Fig. 2). The small bowel was everted; a segment-resection with a side-to-side ileo-ileostomy was performed. The resected bowel was sent to the pathologist. The patient stayed in the normal ward and could then be discharged without complications (Fig. 3).

The pathology showed a 2.5 cm MD containing two NETs measuring 1.6 and 1.5 cm, respectively, with a TNM Classification (UICC 8. Vers. 2017) pT3, Pn0, LVI 0, R0. The results were discussed on our Tumour-Board and a staging via MRT, PET-CT was suggested. A colonscopy was performed to exclude a metacronous bowel tumour. The follow-ups were organized but after an inconspicious colonoscopy, the patient denied all further exams and never appeared again for check-up.

## DISCUSSION

MD occurs in around 2% of the population with a higher prevalence in men than in women. Between 60 and 70% of the patients are asymptomatic, but 6% develop small bowel obstruction during lifetime [1]. NETs have the highest prevalence in MD. The best explanation for this must be searched in the common origin of the MD and the NET from the endodermal and neural crest. Despite a 67% of macroscopic evidence of the disease during surgical resection [1], the 5-year-survival rate of stage I (T1, N0, M0) reaches 80% but 25% of patients already have metastases at the moment of diagnosis [2–5]. The recommendation for the surgical removal is given by NET bigger than 5 mm [1–5]. MRT and PET-CT are usually used to plan the surgical removal for elective operations [5]. Surgical approach represents the gold standard to treat this pathology. The operation can be performed through an open or a laparoscopic access. The open access allows a better operation field and for a curative surgery is more indicated to reach a R0 resection [1–5]. The ENETS Guidelines are suggesting an oncological resection basing on the dimension: for example by an NET of the Appendix if bigger than 2 cm. If there are intraoperative signs of a neoplasm, an oncological removal must be discussed. In case of an emergency surgery, the purpose will be securing a histological diagnosis, to plan the further treatment [5]. The most debated questions in the recent literature are the complication rate of either access and the management of incidentalomas. With techniques like laparoscopy, CT scan, and better preoperative and post-operative management, overall complications were significantly reduced in the last few years. The recent literature reports lower rates of complications, a small number needed-to-treat (NNT) to achieve a clinical advantage and a good outcome through a laparoscopic access [1].

Based on our experience with MD, we believe that the approach with a single incision gives us the possibility to perform a lot of operations without the need to convert to an open approach. This gives us enough space to explore the operation field with a lower risk of developing a ventral hernia. We can strongly recommend this kind of minimal access surgery, for an emergency operation, as a feasible procedure in the everyday routine.
